# Amino acid solution mitigates hypothermia response and intestinal damage following exertional heat stroke in male mice

**DOI:** 10.14814/phy2.15681

**Published:** 2023-05-22

**Authors:** Michelle A. King, Astrid Grosche, Shauna M. Ward, Jermaine A. Ward, Anusree Sasidharan, Thomas A. Mayer, Mark L. Plamper, Xiaodong Xu, Matthew D. Ward, Thomas L. Clanton, Sadasivan Vidyasagar

**Affiliations:** ^1^ Thermal and Mountain Medicine Division United States Army Research Institute of Environmental Medicine Natick Massachusetts USA; ^2^ Radiation Oncology University of Florida College of Medicine Gainesville Florida USA; ^3^ Health and Human Performance University of Florida Gainesville Florida USA

**Keywords:** cytokines, exercise, gastrointestinal, inflammation, permeability, supplementation

## Abstract

Increased gut permeability is implicated in the initiation and extent of the cytokine inflammatory response associated with exertional heat stroke (EHS). The primary objective of this study was to determine if a five amino acid oral rehydration solution (5AAS), specifically designed for the protection of the gastrointestinal lining, would prolong time to EHS, maintain gut function and dampen the systemic inflammatory response (SIR) measured during EHS recovery. Male C57/BL6J mice instrumented with radiotelemetry were gavaged with 150 μL of 5AAS or H_2_O, and ≈12 h later were either exposed to an EHS protocol where mice exercised in a 37.5°C environmental chamber to a self‐limiting maximum core temperature (Tc,max) or performed the exercise control (EXC) protocol (25°C). 5AAS pretreatment attenuated hypothermia depth and length (*p* < 0.005), which are indicators of EHS severity during recovery, without any effect on physical performance or thermoregulatory responses in the heat as determined by percent body weight lost (≈9%), max speed (≈6 m/min), distance (≈700 m), time to Tc,max (≈160 min), thermal area (≈550°C∙min), and Tc,max (42.2°C). EHS groups treated with 5AAS showed a significant decrease in gut transepithelial conductance, decreased paracellular permeability, increased villus height, increased electrolyte absorption and changes in tight junction protein expression pattern suggestive of improved barrier integrity (*p* < 0.05). No differences were witnessed between EHS groups in acute phase response markers of liver, circulating SIR markers, or indicators of organ damage during recovery. These results suggest that a 5AAS improves Tc regulation during EHS recovery through maintaining mucosal function and integrity.

## Graphical abstract

Exertional heat stroke (EHS) is a debilitating and potentially fatal condition for which few prevention strategies exist. These results suggest that a 5AAS improves Tc regulation during EHS recovery through maintaining mucosal function and integrity and that protecting the GI tract during EHS may be beneficial in some of the health consequences of EHS exposure.

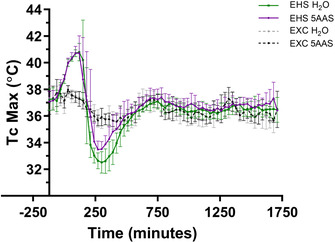

## 1 INTRODUCTION

Intestinal barrier dysfunction is implicated in a myriad of chronic conditions and potentially fatal diseases (Bischoff et al., 2014). While the pathophysiology of exertional heat stroke (EHS) is multifactorial, intestinal barrier dysfunction is suggested to play a significant role in the initiation and severity of this condition (Bouchama & Knochel, 2002; Leon & Bouchama, 2015; Pires et al., 2017).

The translocation of luminal antigens such as bacteria, lipopolysaccharides, or other bacterial components into underlying intestinal tissues and circulation cause local and systemic inflammation. This includes the release of proinflammatory cytokines which can impair mucosal and barrier function. These events are linked to both decrements in physical performance and the extent of organ injury in extreme hyperthermia (Lim et al., 2009; Pals et al., 1985; Vargas & Marino, 2014). Further, exaggerated translocation of bacteria and its toxins is the proposed mechanism responsible for septic conditions following hemorrhagic shock, burn injury, trauma, and irradiation treatments (Baker et al., 1988; Nejdfors et al., 2000; Nunez Lopez et al., 2017; Parrillo, 1993; Schatten et al., 1955). Therefore, an approach that can prevent bacterial translocation by improving intestinal barrier function may be beneficial for the treatment of EHS as well as other conditions that share similar mechanisms of injury (Zhang et al., 2015).

To date, no prophylactic measure exists that can prevent the multiorgan dysfunction that occurs with EHS. Select prophylactics demonstrate improvements in gut barrier dysfunction during exercise, hyperthermia, and sepsis in animal models (Hall et al., 2001; Marchbank et al., 2011; Phillips et al., 2015; Prosser et al., 2004; Pugh et al., 2017; Singleton & Wischmeyer, 2006; Soares et al., 2014; Zuhl et al., 2014). However, many of these interventions did not translate to humans (Bynum et al., 1979; Gathiram et al., 1988; Gathiram, Wells, Brock‐Utne, & Gaffin, 1987; Gathiram, Wells, Brock‐Utne, Wessels, & Gaffin, 1987). Alternatively, amino acid supplementation has proven highly effective at protecting the intestinal barrier in both animal and human models of heat and hypoxia (Marchbank et al., 2011; Soares et al., 2014), but has yet to be tested in EHS. Here, we utilize a five amino acid oral rehydration solution (5AAS), which has been shown to reduce intestinal permeability and suppress endotoxin translocation following radiation exposure, to determine its potential efficacy in an established mouse model of EHS (King et al., 2015). This model was chosen as it closely reflects the responses observed in EHS in humans.

The hypotheses of this study are threefold: (1) a 5AAS will reduce paracellular permeability in the small intestine during exertional heat stress; (2) a 5AAS will delay the onset of EHS by increasing distance run and/or maximal speed attained; and (3) a 5AAS will dampen the systemic inflammatory response, lessen the multi‐organ damage, and decrease recovery time following EHS. If this study supports these hypotheses, it could influence the prevention or treatment of heat illnesses and provide additional insights into the pathophysiology of EHS.

## 2 METHODS

### 2.1 Animal care

A total of 201 C57BL/6J male mice (Jackson Laboratories, Bar Harbor) weighing an average of 27.3 ± 1.7 g and approximately 2.5 months of age were used in this study. Female mice have a separate and unique response to EHS (Garcia et al., 2018) and therefore were not included in the current experimental design. The average number of mice per group was 13, although this varied per experimental group as described below. The total number of experimental groups was 15. This included four groups (EHS 5AAS, EHS H_2_O, EXC 5AAS, EXC H_2_O) each with three time points (30 min, 3 h, and 24 h) plus three naïve control groups (no gavage, 5AAS, and H_2_O). Mice were group‐housed until implantation of temperature telemetry devices. Following surgery mice were individually housed in 29.21 cm × 19.05 cm × 12.7 cm cages lined with Harlan corn cob bedding and maintained on a 12:12 light dark cycle and 20–26°C and 30%–70% relative humidity (RH). A standard chow diet (LM‐485 m Envigo; Teklad, Madison, WI.) and water were provided ad libitum until initiation of the EHS protocol. Experiments were performed in the morning of the light cycle (≈0630–1000) to avoid the potential confounding influence of circadian changes in Tc and mucosal function. This animal protocol was approved by the University of Florida Institutional Animal Care and Use Committee (IRB Approval #201710081) as well as The United States Army Research Institute of Environmental Medicine (IRB Approval #18‐05A). All research conducted in this report adhered to the “Guide for the Care and Use of Laboratory Animals” of the Institute for Laboratory Animal Research, United States National Research Council.

### 2.2 Animal preparation and familiarization

Radio telemetry transmitters (±0.1°C; G2 E‐Mitter, Starr Life Sciences) were implanted into the abdominal cavity under isoflurane anesthesia (4%, 0.4–0.6 L/min O_2_ flow in an induction chamber, followed by continuous anesthesia using a nosecone delivery system (1.5%, 0.6 L/min). During surgical recovery buprenorphine injections were administered at least every 12 h for 48 h as a post‐surgical analgesic. Mice were undisturbed for 1 week following the surgical procedure. During this recovery phase, mice had access to a Mouse House® (Nalgene Nunc Int.) and standard nestlets (2″ × 2″ size, Ancare) as forms of enrichment. On the second week of recovery, running wheels (Starr Life Sciences) were introduced into the cages and mice were allowed to run ad libitum while activity was recorded. Following recovery, four familiarization sessions were conducted in the environmental chamber (3950; Thermo Forma) at ambient temperature (~25 °C) in a forced running wheel (80840; Lafayette Instrument). These sessions consisted of a 1 h incremental protocol where mice were started at an initial speed of 2.5 m/min and increased 0.5 m/min every 10 min for 60 min. No shock or any other manual stimuli were used to maintain running speed.

### 2.3 EHS protocol

Following the last familiarization session, mice were given 2 days of rest with free access to the running wheel in their cages. Mice received either 150 μL (dosing was based on previously published literature (Yin et al., 2014)) of the 5AAS (marketed as enterade®; containing: l‐aspartic acid, l‐valine, l‐serine, l‐threonine, l‐tyrosine; with 1.2 g of protein, 230 mg sodium, 93 mg potassium/237 mL; 142 mOSM) or water (H_2_O) via oral gavage, ≈12 h before the EHS protocol. In preliminary work, this specific combination of amino acids was shown to have the greatest protective effect on the intestinal barrier in‐vitro and therefore was utilized in this experiment. Mice were orally gavaged ≈12 h prior to the experiment as the stress of gavage can increase Tc ~1.5°C and influence thermoregulatory outcomes. Tc was monitored and averaged over 30‐sec intervals. For standardization purposes, heat was initiated when Tc was < 37.5°C (as previously published (King et al., 2015)), then the environmental temperature (*T*_env_) of the chamber was increased to the target of 37.5°C and maintained over the course of the experiment at approximately 37.3 ± 0.6°C, 30 ± 6% RH. Once *T*_env_ of the chamber equilibrated to the target temperature, the mouse was taken from its home cage within the heat chamber and placed into the forced running wheel and the EHS protocol initiated. During the protocol, mice were monitored via camera (IP70; Compro Technology) to minimize disturbance and ensure safety of the animal. Similar to the training protocol, mice began at a speed of 2.5 m/min, which was then increased 0.3 m/min every 10 min until the mouse reached a Tc of 41°C, when running speed was then kept constant. The experimental endpoint was defined as a Tc of 42.7°C or symptom limited by a sudden loss of consciousness and collapse. All mice collapsed from EHS prior to reaching the cut off temperature. Following collapse, the mouse was immediately removed from the chamber, weighed, and returned to its home cage for undisturbed recovery in ambient temperature (24.1 ± 0.1°C, 44 ± 12% RH) until the time of sacrifice (30 min, 3 h, and 24 h post collapse). Separate mice were used for exercise controls (EXC) and were matched for duration and intensity of forced wheel running with *T*_env_ maintained at 26.1 ± 3.1°C and 38 ± 11% RH.

### 2.4 Naïve control groups

To determine the impact of gavage with 5AAS or H_2_O on the variables measured, we included three additional naïve control groups. These groups consisted of one non‐heated control group that did not receive gavage or exercise (NC) (*n* = 4) and two additional non‐heated control groups that received H_2_O (NC H_2_O) (*n* = 6) or 5AAS (NC 5AAS) (*n* = 6) via gavage without exercise. No differences existed among these three groups, and for ease of presentation the NC group is represented in figures. Experiments where only NC H_2_O or NC 5AAS were utilized for comparison (FITC‐Dextran measures, conductance, and current), the NC H_2_O groups are represented as controls.

### 2.5 Thermoregulatory calculations

Thermal area was used as an estimate of thermal stress and calculated as defined by Leon et al. (2005) and described elsewhere (King et al., 2015). Briefly, this equals the area under the curve of the temperature profile for all points at which Tc was >37.5°C (units = °C∙min). Tc minimum is the lowest average value attained over 30 s and hypothermia depth is the lowest 1‐h average Tc during recovery.

### 2.6 Tissue and blood samples

Tissue and blood samples were obtained at 30 min, 3 h, and 24 h after collapse to determine the impact of the 5AAS versus H_2_O on the cytokine inflammatory response and organ damage. The same analyses were also performed on EXC groups to understand the impact of EHS alone. Time points were chosen based on previous studies displaying robust responses at 30 min and 3 h with return to baseline at 24 h (King et al., 2015, 2016). Mice were placed under isoflurane anesthesia for sample collection. Blood samples were obtained via cardiac puncture, placed in heparin or EDTA tubes, used immediately in point of care testing or spun at 3000 RCF for 5 min at 4°C to separate plasma from the buffy coat, divided into aliquots and stored at −80°C for later analysis.

### 2.7 Transepithelial electrical conductance assessment using electrophysiology

Small intestine tissue was harvested after 30 min, 3 h, or 24 h of recovery. Transepithelial electrical conductance (*G*; in mS/cm^2^) and basal short‐circuit current (*I*_sc_; in μeq/h/cm^2^), were recorded and analyzed in ileal mucosa mounted in Ussing chambers as previously described (Soares et al., 2014).

### 2.8 Paracellular permeability assessment using fluorescein isothiocyanate (FITC)‐conjugated dextran

Nonionic particle permeation using fluorescein isothiocyanate (FITC)‐conjugated dextran (4 kDa; Sigma #46944) was used to evaluate the permeability of hydrophilic nonionic tracer macromolecules through the paracellular pore pathway (0.4 nm in radius) of the intestinal barrier. Small intestine tissue was harvested after 30 min, 3 h, and 24 h of recovery. Tissues were mounted in Ussing chambers and FITC‐dextran was added to the mucosal side of the Ussing chambers and allowed to equilibrate for 45 min to let the labeled particles reach a steady‐state rate of flux into the serosal side of the Ussing chamber. Samples were collected from the serosal side and the concentration of FITC‐dextran determined spectrophotometrically, with an excitation at 485 nm (20 nm bandwidth) and an emission of 528 nm (20 nm bandwidth).

### 2.9 Histomorphometric measurements

Biopsy specimens of mid jejunum and ileum were fixed in neutral‐buffered 10% formalin, embedded in paraffin, cut into 4‐μm‐thick cross‐sections (intestinal rings), placed on silane‐coated glass slides, and stained with hematoxylin and eosin stain for examination via light microscopy. For histomorphometric assessment of morphological changes, a computer‐based imaging‐analysis program (Olympus Cell Sense Standard) was used and three intestinal rings from each tissue were examined using the Olympus FV1000 microscope and a 10× objective. Mucosal height was measured by the same investigator as the mean vertical distance between the muscularis mucosa and the lumen surface at the villus tip (Figure 3B). Villus height was the mean vertical distance between the base of each villus and the villus tip calculated from 20 villi in two different mice per group which were randomly selected (Yin et al., 2016). The same villi were used to calculate the mean width measured as a horizontal distance between the lateral surface epithelia at the middle of each villus. The mean crypt height was calculated from maximum vertical distance between the top and the bottom of 20 crypts from two different mice, and the crypt width was measured as the horizontal distance at the middle of each crypt.

### 2.10 Immunofluorescence imaging of tight junction protein expression

To determine the changes in the tight junction protein expression patterns, immunofluorescence stain was performed for occludin, claudin‐1, claudin‐2, claudin‐5, and e‐cadherin in ileal mucosa from various test conditions and treatment groups, as previously described (Gupta et al., 2020). Briefly, tissues were deparaffinated and heat‐pretreated in TintoDeparaffinator Citrate (BioSB #BSB0175) and TintoDeparaffinator Hot Rinse (BioSB #BSB0179), blocked with serum‐free protein block (Dako #X0909) and incubated overnight with 1:100 primary antibody diluted in antibody diluent with background‐reducing components (Dako #S3022) at 4°C. The following antibodies were used: occludin rat mAb (clone 6B8A3, RRID: AB_2819194, Jerrold Turner Lab, Brigham and Women's Hospital), claudin‐1 rabbit pAb (clone MH25, RRID: AB_2533997; Invitrogen #71‐7800), claudin‐2 rabbit pAb (RRID: AB_869174; Abcam #ab53032), e‐cadherin rabbit mAb (clone 24E10, RRID: AB_2291471; Cell Signaling #3195), and claudin‐5 rabbit pAb (RRID: AB_2533157; Invitrogen #34‐1600). After thorough washing in Immuno/DNA washer solution (BioSB #BSB0150), sections were incubated for 1 h at room temperature with the following secondary antibodies: goat anti‐rabbit labeled with Alexa Fluor 647 (Invitrogen #A27040) for claudin‐2 and claudin‐5, goat anti‐rat labeled with Alexa Fluor 488 (Invitrogen #A11006) for occludin, or goat‐anti‐rabbit labeled with Texas Red (Abcam #ab6719) for claudin‐1 and e‐cadherin. Nuclei were stained with DAPI for 15 min, and sections were embedded in Fluoromount‐G (Invitrogen #00‐4958‐02) for imaging. Tissues were analyzed with Olympus laser scanning microscope ”Fluoview FV1000” at 400× magnification (40× objective) using the imaging software “FV10‐ASW 3.1.″

### 2.11 Plasma fatty acid binding protein 2 (FABP‐2) as an indirect measure of gut permeability

Plasma ELISA kits were used for the determination FABP‐2 (Cloud‐Clone) concentrations and were performed according to the manufacturer's instructions.

### 2.12 16S rRNA in plasma

*To determine presence of bacteria in the plasma* RNA was isolated from 50 μL EDTA plasma using the miRNeasy Serum and Plasma Kit (Qiagen) and reverse transcribed into cDNA (37°C for 2 h) using the High‐Capacity Reverse Transcription Kit (Applied Biosystems). cDNA was combined with SYBR Green Master Mix (Applied Biosystems), 0.5 μM of the forward (5′‐CTT GTG CGG GCC CCC GTC AAT TC‐3′) and reverse (5′‐AGA GTT TGA TCC TGG CTC AG‐3′) 16S rRNA primers (IDT), and water in a final reaction volume of 20 μL. Genomic DNA from Escherichia coli (Affymetrix) was used as a positive control. RT‐PCR reactions were performed under the following conditions: hold at 95°C for 10 min, 40 cycles of 95°C for 15 s and 60°C for 1 min (StepOne Plus Real‐Time PCR System; Applied Biosystems).

### 2.13 RNA isolation, reverse transcription, and real time PCR

To determine the acute phase response in the liver, RNA extraction and analyses were conducted as described previously (Leon et al., 2013). Briefly, flash frozen tissue was homogenized using the FastPrep‐24 (MP Biomedicals). RNA was isolated from homogenized liver tissue using the RNA Tissue Kit II/QuickGene‐Mini80 (AutoGen). RNA quantity and quality were assessed using a Nanodrop 8000 spectrophotometer (Nanodrop Products). RNA was reverse transcribed into cDNA (37°C for 2 h) using the High‐Capacity Reverse Transcription Kit (Applied Biosystems). TaqMan Gene Expression Assays for target genes (serum amyloid 1 (SAA1), serum amyloid 3 (SAA3), mannose binding lectin (MBL), alpha 2 macroglobulin (A2M), C‐reactive protein (CRP)) in liver were combined with cDNA, Fast Advanced Master Mix, and water in a final reaction volume of 20 μL. RT‐PCR reactions were performed in duplicate under the following conditions: hold at 95°C for 20 s, 40 cycles of 95°C for 1 s and 60°C for 20 s (StepOne Plus Real‐Time PCR System; Applied Biosystems) with >90% efficiency for all genes.

### 2.14 Plasma cytokine measurements

Plasma cytokine and chemokine measurements were determined via Luminex, using MILLIPLEX MAP Mouse cytokine/chemokine pre‐mixed 25 plex assay kits which included the antibodies for the following analytes: granulocyte colony‐stimulating factor (G‐CSF), granulocyte‐macrophage colony stimulating factor (GM‐CSF), Interferon gamma (IFN‐γ), IL‐1α, IL‐1β, IL‐2, IL‐4, IL‐5, IL‐6, IL‐7, IL‐9, IL‐10, IL‐12 (p40), IL‐12 (p70), IL‐13, IL‐15, IL‐17, interferon γ induced protein 10, keratinocyte chemoattractant (KC), monocyte chemoattractive factor‐1 (MCP‐1), macrophage inflammatory protein (MIP) 1α, MIP‐1β, MIP‐2, regulated on activation, normal T cell expressed and secreted (RANTES), and tumor necrosis factor (TNF)α. This assay was performed according to the manufacturer's instructions (MilliporeSigma), as described elsewhere (Welc et al., 2012).

### 2.15 Metabolic hormone measurements of the gut

Plasma metabolic hormones of the gut including gastric inhibitory peptide (GIP), glucagon like‐ peptide 1 (GLP‐1), and pancreatic peptide YY (PYY) were determined as a part of a larger metabolic panel via Luminex not reported here. This was completed using MILLIPLEX MAP Mouse metabolic hormone pre‐mixed 14 plex assay kits which included the antibodies for the following analytes: amylin (active), c‐peptide, ghrelin, GIP, GLP‐1, glucagon, IL‐6, insulin, leptin, MCP‐1, pancreatic polypeptide (PP), PYY, resistin, and TNF‐α. This assay was performed according to the manufacturer's instructions (MilliporeSigma). Values reported for IL‐6, MCP‐1, and TNFα were taken from the above plasma cytokine/chemokine panel to avoid duplication.

### 2.16 Blood biomarkers of multi‐organ dysfunction

Blood biomarkers of multi‐organ dysfunction were measured with whole blood via the Abaxis VETSCAN®VS2 Chemistry Analyzer using the Equine Profile Plus point of care panel with the following variables reported: blood urea nitrogen (BUN) (kidney), creatine kinase (CK) (muscle), and aspartate aminotransferase (AST) (liver).

### 2.17 Statistical analyses

Statistical analyses were performed using GraphPad Prism (La Jolla), SAS JMP (Cary) and OriginPro 2016 (Northampton). Variables were tested for normality; those that were normally distributed are represented as means ± SD. Non‐normally distributed data are presented as medians ± 25%–75% quartiles. An unpaired *t‐*test was used to determine differences between physical, performance, and thermoregulatory characteristics of EHS 5AAS and H_2_O. ANOVA was used for multiple comparisons with Tukey's Test for post hoc comparisons when data were normally distributed and Kruskal–Wallis or Wilcoxon were used for non‐parametric comparisons. Significant fold change in mRNA acute phase proteins were determined by using a one sample Wilcoxon Test. For conductance, FITC‐dextran permeation and J_net_ (Na^+^) Kruskal Wallis was used for multiple comparison and Mann–Whitney Test for post hoc comparison. For histomorphometric measurements, ANOVA was used with Bonferroni corrections for post hoc comparison. Sample size was calculated utilizing Tc as the primary variable with an expected difference between means of 0.5°C and an expected deviation of 0.3°C using a conventional alpha (0.05) and beta (0.20) value. For ease of presentation, groups with similar findings have been summarized by *p* value groupings (*p* < 0.05, *p* < 0.001, *p* < 0.0001). Individual *p* values can be found in the statistical summary table (Table S1).

## 3 RESULTS

### 3.1 Exercise performance and thermoregulatory responses in the heat

**FIGURE 1:**
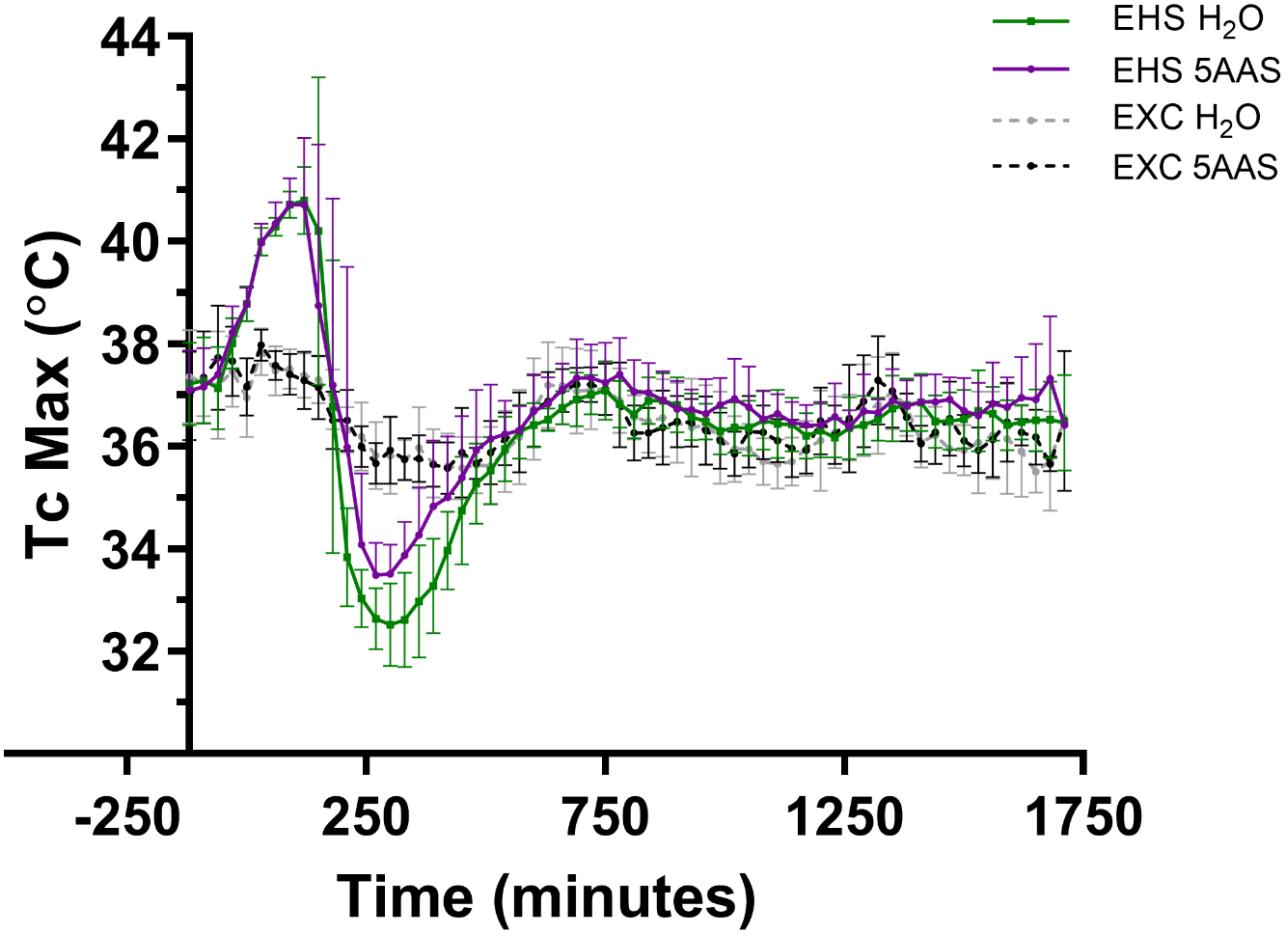
Core temperature responses of EHS and EXC mice administered either 5AAS or H_2_O. Thirty‐minute core temperature (Tc) averages are shown with mean ± SD. Only mice in the 24‐h group are included here; EHS 5AAS (*n* = 15) EHS H_2_O (*n* = 15) EXC 5AAS (*n* = 12) EXC H_2_O (*n* = 14).

Physical performance in the heat as determined by percent dehydration, max speed, and distance were similar between treatment groups (Table 1). Thermoregulatory responses in the heat such as Tc,max and thermal load were nearly identical between treatment groups (*p* > 0.05) (Table 1). Interestingly, the extent of hypothermia during recovery, a marker of EHS severity, was significantly mitigated with 5AAS (Table 1, Figure 1). Minimum Tc (5AAS: 32.9°C ± 0.4; H_2_O: 32.1°C ± 0.8; *p* = 0.002), hypothermia depth (5AAS: 33.3 ± 0.5 °C∙min; H_2_O: 32.5 ± 0.8 °C∙min; *p* = 0.002), and hypothermia length (5AAS: 186.9 ± 47.3 min; H_2_O: 243.6 ± 42.3 min; *p* = 0.002) were all significantly improved by 5AAS (Table 1). 5AAS had no effect on thermoregulatory responses or physical performance in EXC groups (*p* > 0.05).

**TABLE 1:** Effect of 5AAS or H_2_O administration on physical performance and thermoregulatory responses during EHS and recovery.

	EHS 5AAS	EHS H_2_O	***p*** value
Weight			
Initial weight (g)	27.8 ± 1.7	27.3 ± 1.4	0.1027
Performance			
Max speed (m/min)	5.9 ± 1.1	5.8 ± 0.1	0.5742
Distance (m	707.8 ± 251.5	693.9 ± 180	0.7595
Thermoregulatory response			
Body weight lost (%)	9.4 ± 2.1	9.3 ± 1.7	0.8177
Tc max (°C)	42.2 ± 0.2	42.2 ± 0.2	0.9673
Time to Tc max (min)	160.9 ± 38.8	155.5 ± 27.7	0.8463
Thermal load (°C min)	552.5 ± 89.5	545.7 ± 82.7	0.7098
Ascending thermal area (°C min)	523.7 ± 88.2	519.0 ± 79.8	0.7885
Descending thermal area (°C min)	26.9 ± 6.3	26.5 ± 5.5	0.7739
Hypothermia severity			
Time to Tc minimum (min)	129.3 ± 34.6	136.2 ± 39.7	0.6159
Tc minimum (°C)	32.9 ± 0.4	32.1 ± 0.8	0.0020**
Hypothermia depth (°C)	33.3 ± 0.5	32.5 ± 0.8	0.0024**
Hypothermia length (min)	186.9 ± 47.3	243.6 ± 42.3	0.0017**

*Note*: 5AAS (*n* = 48) or H_2_O (*n* = 46). Hypothermia severity measures were taken from mice that were allowed to recover for 24 h following EHS 5AAS or H_2_O (*n* = 15 each). Mean ± SD and exact *p* values are shown, unpaired *t*‐test.

** *p <* 0.01.

The length and duration of hypothermia is commonly used as an indicator of the severity of heat stress imposed. However, hypothermia depth and length did not correlate with Tc,max or the ascending thermal area in either EHS group. Further, no strong correlations between any of the circulating biomarkers (as discussed below in Section 3.2.5) and Tc,max were found irrespective of treatment group.

### 3.2 Gut permeability and barrier function

#### 3.2.1 Changes in small intestinal transepithelial electrical conductance following EHS

**FIGURE 2:**
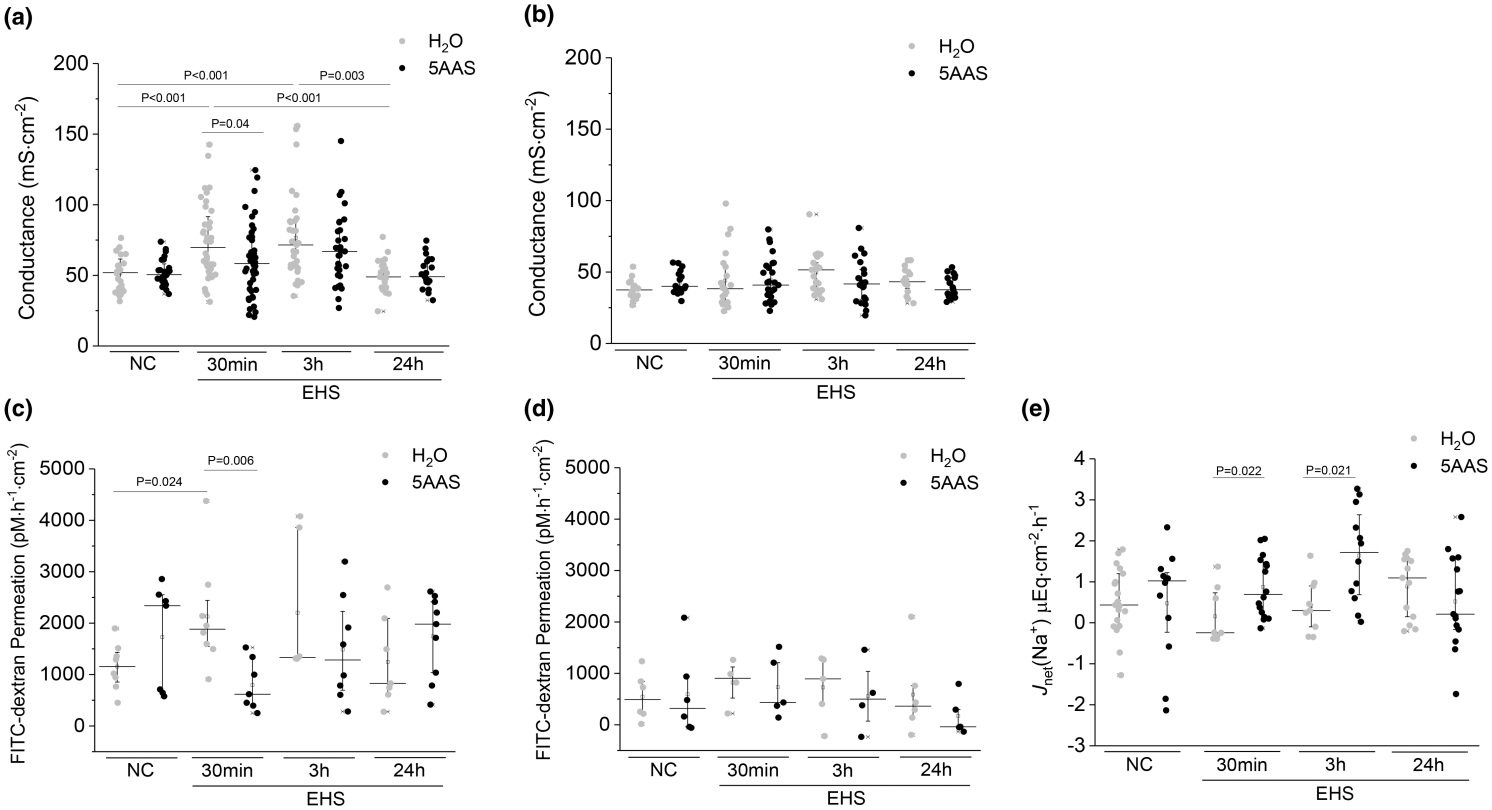
Effect of 5AAS on conductance, FITC‐dextran permeability and net Na^+^ flux in mouse small intestine following EHS. (a) Changes in conductance in jejunal mucosa significantly increased values following EHS that was attenuated by 5AAS treatment (*n* = 24 to 44). (b) In ileal mucosa, conductance did not change with EHS or treatment (*n* = 16 to 27). (c) 4 kDa FITC‐dextran permeation in jejunal mucosa was significantly increased in mice following EHS that was attenuated by 5AAS treatment (*n* = 6 to 9). (d) 4 kDa FITC‐dextran permeation in ileal mucosa did not change with EHS or 5AAS treatment (*n* = 4 to 6). (e) Changes in net Na^+^ flux demonstrate decreased Na^+^ absorption at 30 min and 3 h post EHS in water‐treated mice, while treatment with 5AAS preserve a normal or increased Na^+^ absorption at 30 min or 3 h post EHS respectively (*n* = 8 to 19). Group averages are shown as median ± IQR. Kruskal–Wallis followed by MannWhitney Test were used for multiple and post hoc comparison (*p* < 0.05).

The presence of 5AAS in the Ringer's solution bathing NC jejunal and ileal tissues mounted in Ussing chambers did not change the transepithelial electrical conductance (NC H_2_O 52.0 ± 9.6 mS cm^−2^ vs. NC 5AAS 51.3 ± 12.9 mS cm^−2^; *n* = 24, and 42.6 ± 1.9 mS cm^−2^ vs. 37.4 ± 1.7 mS cm^−2^; mean ± SD; *n* = 24). In the jejunum, EHS resulted in a significant increase in conductance at 30 min and 3 h recovery time (NC H_2_O vs. 30 min or 3 h EHS H_2_O; *p* < 0.001 or *p* < 0.001; *n* = 36 or 33) (Figure 2A). Conductance returned to normal naïve control levels at 24 h (*n* = 23). 5AAS treatment resulted in a significant decrease in conductance compared to corresponding tissues treated with water at 30 min (30 min EHS 5AAS vs. 30 min EHS H_2_O; *p* = 0.04; *n* = 44). At 3 h and 24 h into recovery following EHS, no significant differences in conductance were observed in jejunal tissue between 5AAS and corresponding water‐treated groups (EHS H_2_O vs. EHS 5AAS) (Figure 2A). In the ileum, conductance did not change post EHS in comparison to NC when treated with water or 5AAS (Figure 2B). EXC (any group) were not different between groups in comparison to NC H_2_O or NC 5AAS treatment group (*p* > 0.05). These results suggest that EHS induced a significant increase in conductance only in the jejunum and not in the ileum. 5AAS decreased jejunal mucosal conductance as early as 30 min, and conductance returned to basal control levels by 24 h following EHS (Figure 2A,B).

At 30 min post EHS, mice treated with water showed a significant increase in 4 kDa FITC‐dextran permeation in jejunal mucosa when compared to NC H_2_O (30 min EHS H_2_O 2128.6 ± 1051.5 vs. NC H_2_O 1154.4 ± 454.6 pm·h^−1^·cm^−2^; mean ± SD; *p* = 0.024; *n* = 8) (Figure 2C). Treatment with 5AAS showed a significantly lower FITC‐dextran permeation at 30 min post EHS when compared to corresponding water‐treated mice (30 min EHS H_2_O vs. 30 min EHS 5AAS; *p* = 0.006; *n* = 7 tissues) (Figure 2C). There were no significant changes at 3 h or 24 h post EHS, in FITC‐dextran permeation when 5AAS‐treated mice were compared to the water‐treated group. EHS or oral treatments did not significantly change tissues isolated from the ileum (Figure 2D).

#### 3.2.2 Changes in small intestinal transepithelial Na^+^ absorption following EHS

No difference in net Na^+^ absorption (*J*_net_Na) was observed in NC H_2_O versus NC 5AAS (Figure 2E). EHS resulted in a small but non significant decrease in *J*_net_Na at 30 min in EHS H_2_O when compared to NC H_2_O. At 30 min, treatment with 5AAS significantly increased *J*_net_Na when compared to the corresponding water‐treated group (30 min EHS H_2_O vs. EHS 5AAS; *p* = 0.022; *n* = 8–16 tissues). Similarly, at 3 h recovery time following EHS, tissues from 5AAS‐treated mice showed a significant increase in *J*_net_Na when compared to the corresponding water‐treated group (3 h EHS H_2_O vs. EHS 5AAS; *p* = 0.021; *n* = 9–12 tissues). At 24 h recovery from EHS, small intestinal tissues did not show any differences in *J*_net_Na regardless of treatment when compared to the corresponding naïve controls (Figure 2E). These results suggest that 5AAS increased Na^+^ absorption during recovery from EHS.

#### 3.2.3 Changes in intestinal histomorphology and histomorphometry following EHS

**FIGURE 3:**
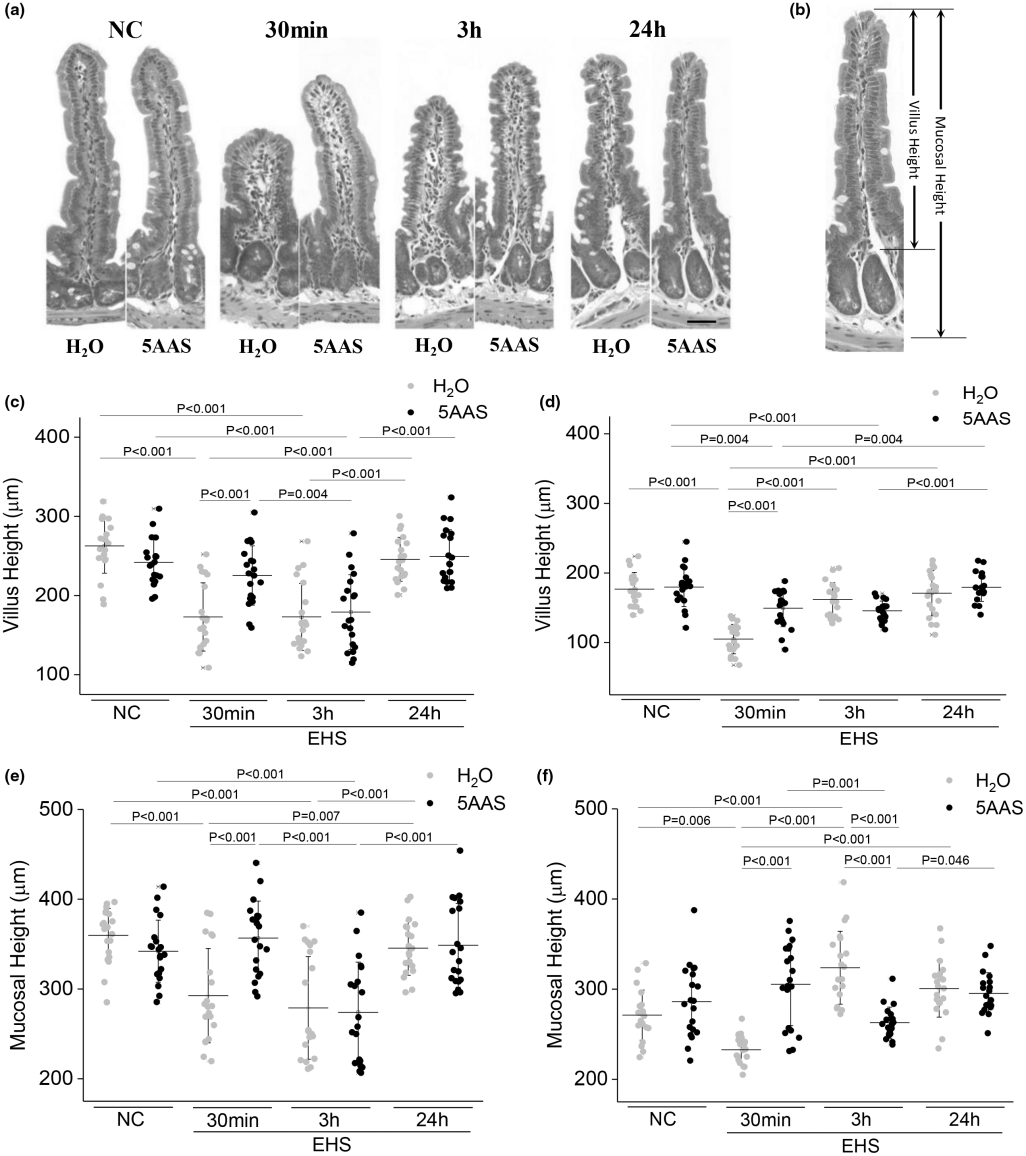
(a) Representative microphotographs of histomorphological changes of jejunal mucosa in mice before, and 30 min, 3 h and 24 h after EHS demonstrate decreased mucosal height and villus height 30 min post EHS. These changes returned to control levels within 24 h (H&E, bar = 30 μm) (b) Representative image showing measurements used to determine villus and mucosal height in mouse jejunum. (c) Changes of villus height in jejunal tissues after treatment with water and 5AAS in mice following EHS. Villus height decreased at 30 min and 3 h post EHS in water‐treated mice while villus height remained within normal range with 5AAS at 30 min when compared to NC. Group averages are shown as mean ± SD. ANOVA followed by Bonferroni Test were used for multiple and post hoc comparison (*p* < 0.05); *n* = 20. (d) In ileum, changes in villus height were similar to the jejunum characterized by decreased values 30 min and 3 h post EHS H_2_O, while mice had longer villi in 30 min EHS 5AAS. Changes of mucosal height in jejunal (e) and ileal tissues (f) followed similar pattern as the villus height. Group averages are shown as mean ± SD. ANOVA followed by Bonferroni test were used for multiple and post hoc comparison (*p* < 0.05); *n* = 20.

The effect of EHS on villus and mucosal height was measured in two mice per group. Representative images of mucosal and villus height are shown in Figure 3A,B. Villus height did not differ in jejunal naïve control tissues from mice treated with water (NC H_2_O) or 5AAS (NC 5AAS; Figure 3C). During 30 min recovery from EHS, villus height was significantly decreased in water‐treated tissues when compared to corresponding naïve controls (EHS H_2_O vs. NC H_2_O; *p* < 0.001; *n* = 20 villi). Treatment with 5AAS resulted in a significant increase in villus height when compared to the corresponding water‐treated group (30 min EHS H_2_O vs. 30 min EHS 5AAS; *p* < 0.001; *n* = 20). Three‐hour recovery time showed a significant decrease in villus height in both water and 5AAS‐treated tissues following EHS when compared to naïve controls (NC H_2_O vs. 3 h EHS H_2_O, and NC 5AAS vs. 3 h EHS 5AAS; *p* < 0.001). Villus height recovered in water and 5AAS‐treated groups at 24 h to values seen in naïve controls (24 h EHS H_2_O vs. NC H_2_O, and 24 h EHS 5AAS vs. NC 5AAS; Figure 3C). In the ileum, tissues from 30 min post EHS (both H_2_O and 5AAS) showed a decrease in villus height when compared to corresponding naïve controls (30 min EHS H_2_O vs. NC H_2_O; *p* < 0.001; and 30 min 5AAS vs. NC 5AAS; *p* = 0.004; *n* = 20), as shown in Figure 3D. But villi remained longer after 5AAS treatment at 30 min when compared to the corresponding water‐treated group (30 min EHS 5AAS vs. 30 min EHS H_2_O; *p* < 0.001; *n* = 20). Villus height recovered in the water‐treated group at 3 h while villus height of the 5AAS‐treated group returned to NC values at 24 h post EHS (Figure 3D). Mucosal heights paralleled changes in villus height in control, 30 min, 3 h, and 24 h in both, ileal and jejunal tissues (Figure 3E,F).

#### 3.2.4 Effect of 5AAS on intestinal tight junction protein expression following EHS

Because EHS resulted in a significant increase in FITC‐dextran permeation in the jejunum and not in the ileum, we evaluated changes in tight junction protein expression in jejunal tissues for 30 min, 3 h, and 24 h periods following EHS.

**FIGURE 4:**
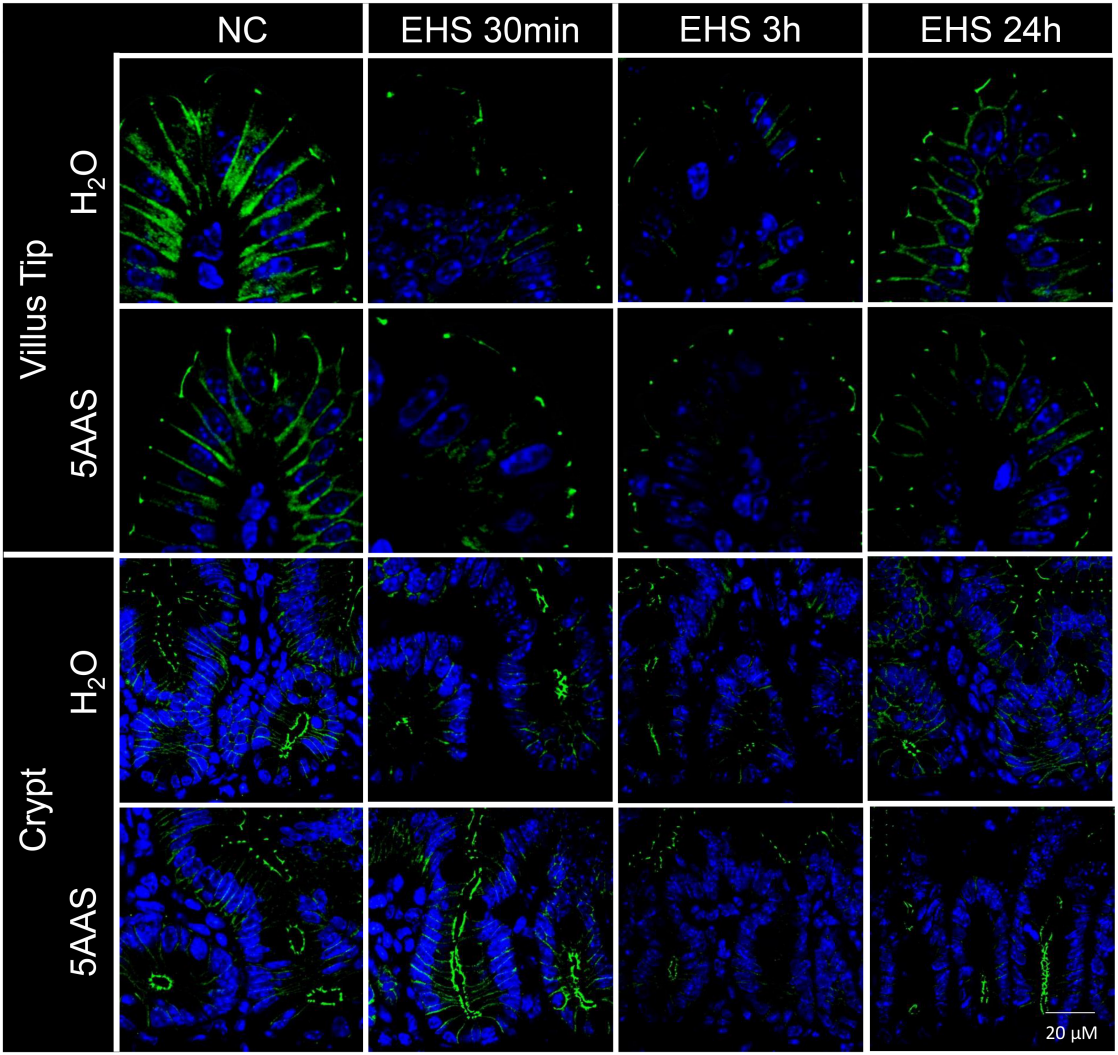
Representative immunofluorescence images of longitudinal jejunal sections showing occludin expression in apical villi that were decreased in both, water and 5AAS‐treated mice at 30 min recovery time (30 min EHS H_2_O and 30 m EHS 5AAS). Occludin expression continued to stay at a lower intensity for up to 3 h post EHS with a more pronounced decrease in mice treated with water compared (3 h EHS H_2_O) to 5AAS (3 h EHS 5AAS). Occludin expression increased at 24 h in both water and 5AAS‐treated mice but did not recover to the level of naïve control tissues (NC). In crypts, treatment with 5AAS showed clear occludin expression in apical tight junctions and basolateral regions of epithelial cells (NC). Occludin expression was relatively lower in water‐treated mice 30 min following EHS (30 min EHS H_2_O), and continued to decrease at 3 h recovery time. Expression levels also decreased in mice treated with 5AAS at 3 h post EHS (3 h EHS 5AAS), however expression levels remained relatively higher when compared to the water‐treated group [occludin (green), nuclei (blue)].

*Occludin*. Occludin expression was seen apically around the tight junctions and along the basolateral membrane of villi and crypts in NC H_2_O and NC 5AAS (representative images that were typical of the observed immunofluorescence are displayed in Figure 4). Representative images depict similar occludin expression in both villi and crypt cell regions in naïve controls treated with water or 5AAS. Representative images suggest that EHS decreased occludin expression at the tight junctions, particularly along the basolateral membranes in villi and at all time points post EHS H_2_O. However, treatment with 5AAS seemed to preserve occludin expression at the tight junctions, but expression along the basolateral membrane was decreased at 30 min (30 min EHS 5AAS) in both, villi and crypts. At 3 h, occludin expression was further reduced in villi and crypts in both treatment groups, however 5AAS‐treated villi presented greater occluding immunofluorescence at the tight junctions (3 h EHS 5AAS). At 24 h, both water‐and EHS‐treated mice showed expression of occludin at tight junctions and along the basolateral membranes. The immunofluorescent intensity was still lower in both treatment groups at 24 h when compared to NC (Figure 4).

**FIGURE 5:**
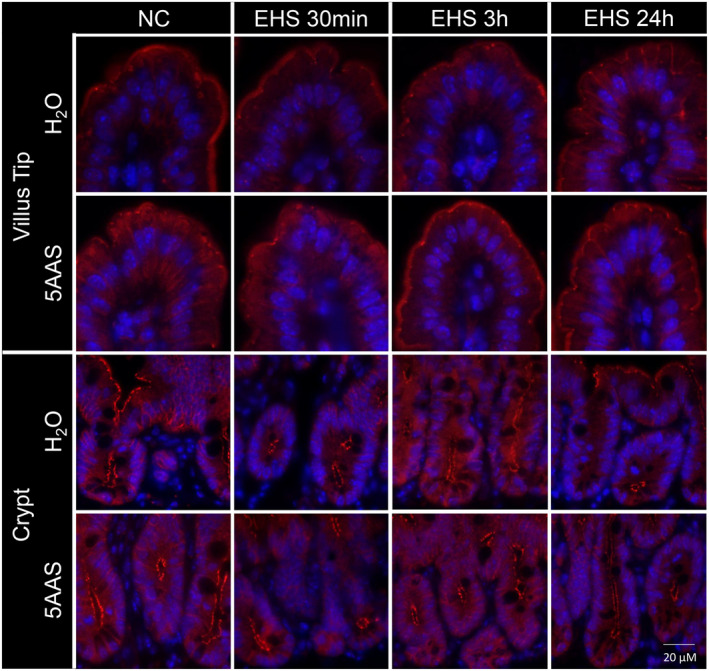
Representative immunofluorescence images of longitudinal jejunal sections showing claudin−1 expression. In apical villi, claudin−1 expression decreased at 30 min and 3 h following EHS in water‐treated mice (30 min and 3 h EHS H_2_O). Claudin−1 expression pattern were slightly decreased at 30 min post EHS in mice treated with 5AAS (30 min EHS 5AAS), but expression levels increased at 3 h and 24 h post EHS with 5AAS treatment. In the crypts, claudin−1 expression was slightly decreased following EHS at each time point in both treatment groups (H_2_Oand 5AAS) however claudin−1 signals were better preserved with 5AAS (30 min, 3 h, 24 h EHS) [claudin−1 (red), nuclei (blue)].

*Claudin‐1*. Claudin‐1 showed a similar expression pattern as occludin with a reduction in its expression levels with EHS that was more pronounced in the water‐treated group (30 min and 3 h EHS H_2_O) (representative images that were typical of the observed immunofluorescence are displayed in Figure 5). The 5AAS‐treated mice showed a better preservation of claudin‐1 expression in villi and crypts at both time points. At 24 h, claudin‐1 expression increased in both water‐and 5AAS‐treated mice. In crypts, claudin‐1 signals were slightly decreased at each time point following EHS in both treatment groups however claudin‐1 expression was better preserved with 5AAS at 30 min, 3 h or 24 h post EHS when compared to the corresponding water‐treated animals (Figure 5).

**FIGURE 6:**
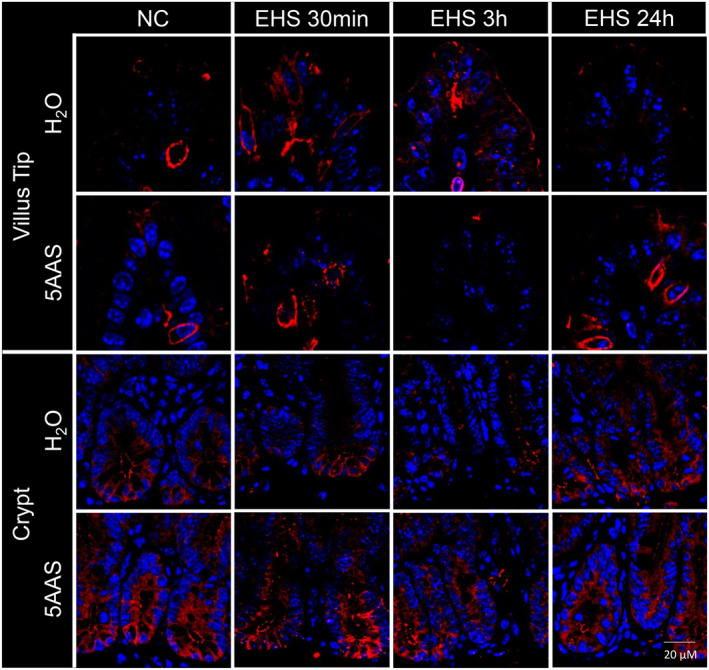
Representative immunofluorescence images of longitudinal jejunal sections showing expression levels of claudin −2, a pore‐forming claudin. In villi of NC mice, claudin −2 expression was negligible, however expression levels increased following EHS (30 min and 3 h EHS H 2O). Claudin −2 expression was increased at the basolateral membranes of villus tips at 30 min post EHS in the water group (30 min EHS H 2O) with further expansion to the apical tight junction regions at 3 h (3 h EHS H 2O). No apparent increase of claudin −2 was observed within the villi in 5AAS‐treated mice. In the crypts of NC mice, claudin −2 expression was evident within the apical and basolateral regions of epithelial cells in both treatment groups (H_2_Oand 5AAS), but expression levels decreased at 30 min and 3 h following EHS. Treatment with 5AAS resulted in higher claudin −2 expression pattern within the crypts compared to the water‐treated groups (30 min and 3 h EHS 5AAS) [claudin −2 (red), nuclei (blue)].

*Claudin‐2:* Immunofluorescence signals for claudin‐2 were mostly absent in the villi of control tissues from water and 5AAS‐treated mice (NC H_2_O and NC 5AAS) (representative images that were typical of the observed immunofluorescence are displayed in Figure 6), but claudin‐2 was strongly expressed in the apical and lateral membrane regions of crypt epithelial cells in NC. EHS resulted in increased claudin‐2 expression within basolateral regions of villi at 30 min (30 min EHS H_2_O), and in the apical and basolateral regions of villi at 3 h (3 h EHS H_2_O), but not in the 5AAS‐treated groups. At 24 h, claudin‐2 signals disappeared from villi in both the 5AAS‐and water‐treated groups (24 h EHS H_2_O and 24 h EHS 5AAS). In the crypts, claudin‐2 expression decreased with EHS at 30 min and 3 h in H_2_O groups, but not with 5AAS. Claudin‐2 expression was restored in both H_2_O and 5AAS‐treated mice at 24 h (Figure 6).

**FIGURE 7:**
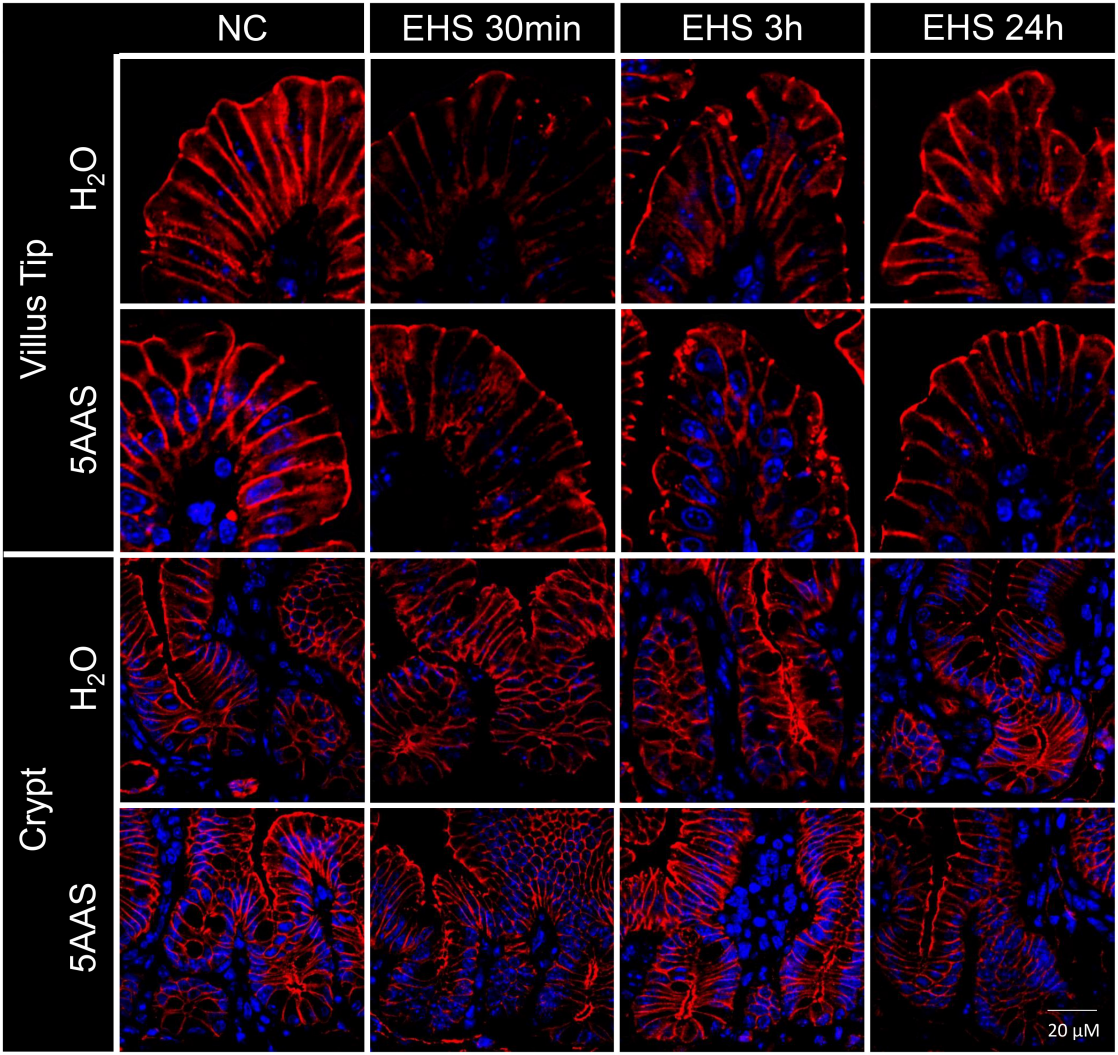
Representative immunofluorescence images of longitudinal jejunal sections showing claudin−5 expression along the tight junctions and basolateral regions of villus and crypt epithelial cells. In villi, claudin−5 expression decreased at 30 min post EHS, and this pattern was more prominent in the water‐treated group. Claudin−5 expression levels recovered over time in both treatment groups. In the crypt cell region, claudin−5 expression levels were higher in control mice treated with 5AAS. EHS resulted in a marginal decrease of claudin−5 expression within crypts in water‐treated mice while expression levels remained unchanged with 5AAS [claudin−5 (red), nuclei (blue)].

*Claudin‐5*. Claudin‐5 expression was seen along apical tight junctions and the basolateral membrane of villi in both NC 5AAS and H_2_O control tissues (representative images that were typical of the observed immunohistochemistry are displayed in Figure 7). A similar expression pattern was also seen in the crypts. EHS decreased claudin‐5 expression in the basolateral regions of villus epithelial cells in EHS H_2_O mice at 30 min, while its expression was largely unaltered in the 30 min EHS 5AAS‐treated group. Claudin‐5 expression levels remained unchanged in villi at 3 h and 24 h post EHS in both H_2_O and EHS 5AAS. In the crypt cell region, claudin‐5 expression levels were higher in NC 5AAS‐treated mice, and EHS decreased expression pattern marginally in 30 min EHS H_2_O mice while crypt claudin‐5 expression remained unaltered with 5AAS at all time points (Figure 7).

*Epithelial (E)‐cadherin*. E‐cadherin expression was seen at the basolateral membranes along the entire length of the villi in both, water‐ and 5AAS‐treated control groups (NC H_2_O and NC 5AAS). E‐cadherin expression remained mostly unaltered at 30 min, 3 h and 24 h recovery time after EHS in both treatment groups villi and crypts.

#### 3.2.5 Systemic inflammatory response

*Plasma 16 s rRNA as a measure of circulating bacteria*. Circulating 16 s rRNA was undetected in any group or recovery time point regardless of treatment. Amplification was detected in DNA standards prepared from Escherichia coli DNA.

**FIGURE 8:**
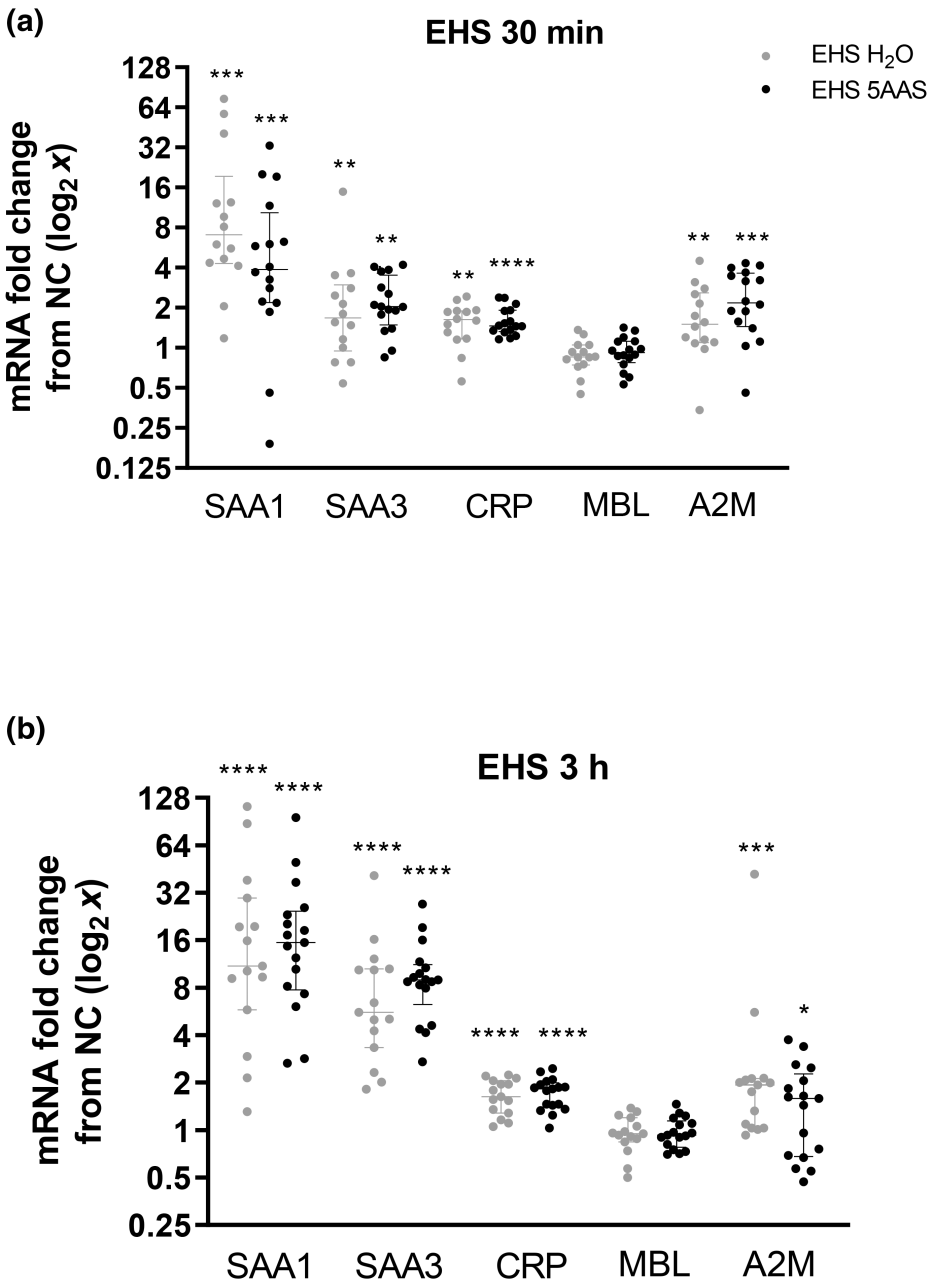
Liver Acute Phase RNA following either 30 min (a) or 3 h (b) recovery from EHS. Fold change in liver acute phase protein mRNA. Serum amyloid A1 (SAA1), serum amyloid A3 (SAA3), C reactive protein (CRP), and alpha‐2‐macroglobulin (A2M) significantly increased following EHS at 30 m and 3 h of recovery. Mannose binding lectin (MBL) was not different from NC at either time point. There were no differences between treatment groups. All changes reported are relative to naïve control (NC) mouse liver (one sample Wilcoxon test). *p <* 0.05 (*), 0.01 (**), 0.001 (***), and <0.0001(****). Exact *p* values can be found in the statistical summary. Benjamini‐Hochberg procedure for multiple ANOVAs false discovery rate (FDR) 20%. (Ns) for SAA1, SAA3, CRP, MBL, and A2M are as follows: EHS 5AAS 30 (16); EHS H_2_O 30 (14); EHS 5AAS 3 (17); EHS H_2_O 3 (15).

*Liver Acute Phase Protein Gene Expression*. EHS significantly increased liver gene expression of SAA1, SAA3, CRP, and A2M at both 30 min and 3 h of recovery (Figure 8A,B) (*p* < 0.05). Peak upregulation of SAA1 (≈25 fold) and SAA3 (≈9 fold) were attained at 3 h of recovery. Furthermore, EHS caused minor upregulation of both CRP (≈2 fold) and A2M (≈2 fold) at both 30 min and 3 h. The 5AAS treatment had no effect on mRNA expression of these markers at any time point (Figure 8A,B). Gene expression in EXC groups was not measured.

**FIGURE 9:**
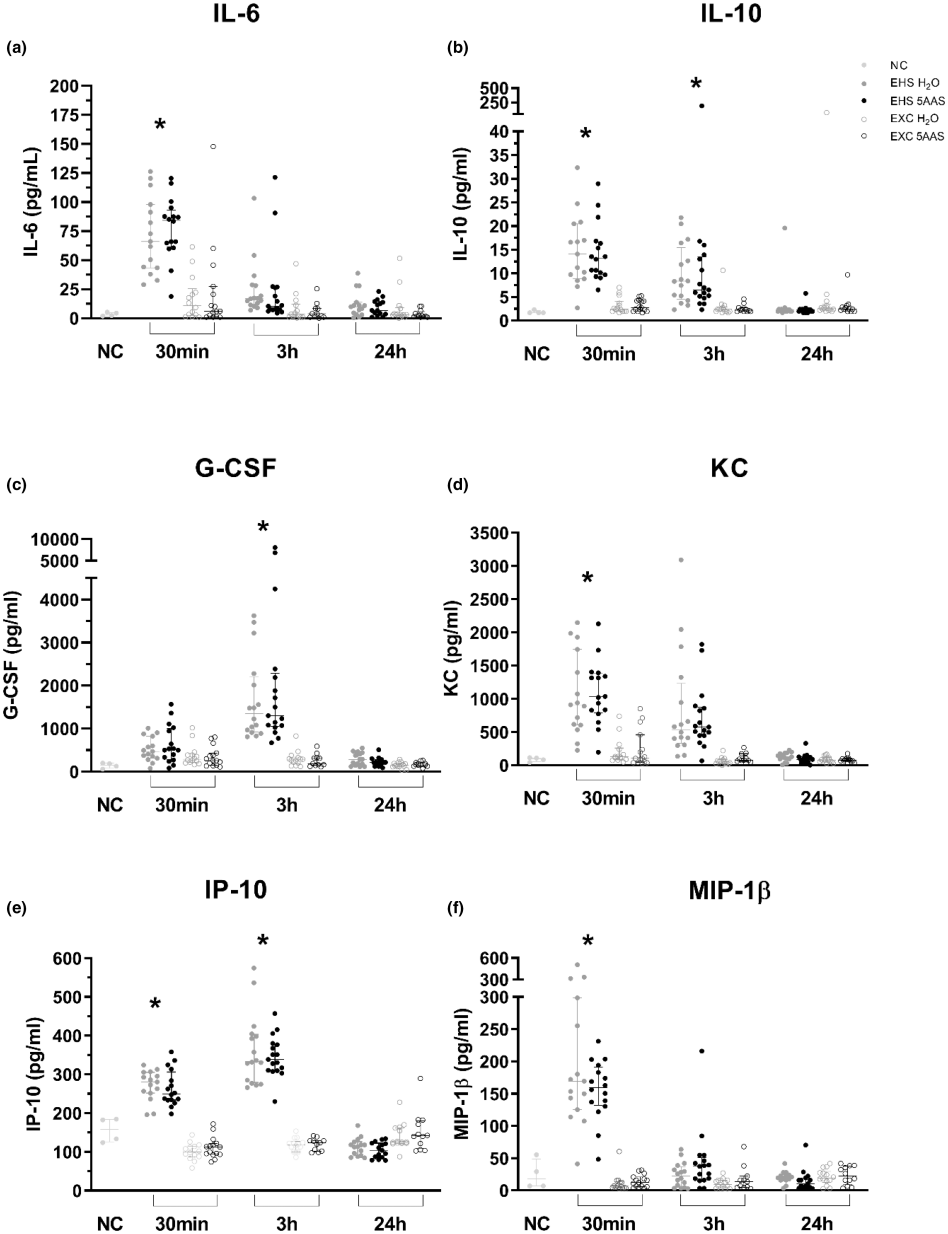
(**A‐F**) Cytokine and chemokine response to EHS at 30 min, 3 h, and 24 h of recovery for Il‐6 (a) IL‐10 (b), G‐CSF (c), KC (d), IP‐10 (e) and MIP‐1β (f). (*) Indicates both 5AAS and H_2_Oare significantly increased from both NC, EXC 5AAS and EXC H_2_O. ANOVA (with Tukey's post hoc) or Kruskal Wallis (with Dunn's post hoc) (*p* < 0.0001) see statistical summary for individual post hoc comparison p‐values. Benjamini‐Hochberg procedure for multiple ANOVAs false discovery rate (FDR) 20%. (Ns) are as follows: EHS 5AAS 30 m (15), 3 h (16), 24 h (15); EHS H_2_O 30 m (16), 3 h (17), 24 h (15): EXC 5AAS 30 m (15), 3 h (11), 24 (12); EXC H_2_O 30 m (14), 3 h (14), 24 (14); NC (4); NC 5AAS (12); NC H_2_O (11).

Congruently, 5AAS did not alter the typical cytokine and chemokine profile response to EXC or EHS (25 plex Luminex panel) at any time point (*p* > 0.05). EHS responsive cytokines and chemokines included IL‐6, IL‐10, G‐CSF, KC, IP‐10, and MIP1‐β, where both 5AAS and H_2_O EHS groups were significantly elevated compared to both NC and EXC groups regardless of treatment (Figure 9A–F) (*p* < 0.05). Of these analytes, IL‐6, IL‐10, KC, and MIP‐1β displayed peak levels at 30 min following EHS. This elevation was sustained in IL‐10 until 3 h of recovery (Figure 9B). G‐CSF and IP‐10 displayed late phase peak responses at 3 h (Figure 9C,E). EXC responses were similar to NC groups, regardless of treatment. Cytokine and chemokine patterns were in concurrence with previously published literature (Garcia et al., 2018; King et al., 2016).

#### 3.2.6 Multi‐organ dysfunction

**FIGURE 10:**
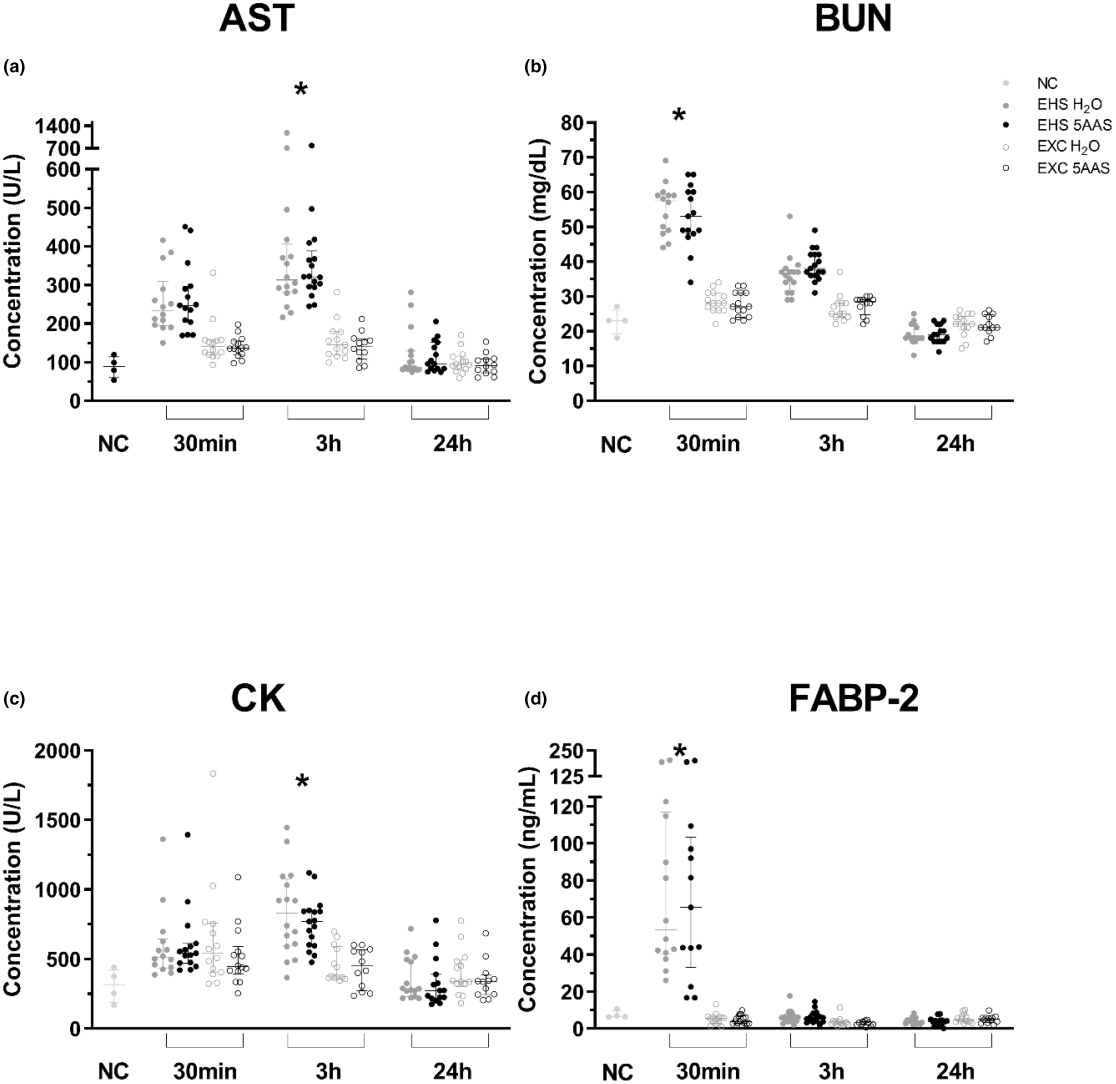
Multi‐organ response to EHS at 30 m, 3 h, and 24 h of recovery for Aspartate Aminotransferase (AST) (a), Blood Urea Nitrogen (BUN) (b), Creatine Kinase (CK) (c), and Fatty Acid Binding Protein−2 (FABP‐2) (D). (*) Indicates both 5AAS and H_2_O are significantly increased from both NC and EXC 5AAS and H_2_O; ANOVA (with Tukey's post hoc) or Kruskal Wallis (with Dunn's post hoc) (*p* < 0.0001) see statistical summary for individual post hoc comparison p values. Benjamini‐Hochberg procedure for multiple ANOVAs false discovery rate (FDR) 20%. (Ns) are as follows for AST, BUN, and CK: EHS 5AAS 30 m (15), 3 h (17), 24 h (15); EHS H_2_O 30 m (14), 3 h (16), 24 h (15): EXC 5AAS 30 m (15), 3 h (11), 24 (15); EXC H_2_O 30 m (14), 3 h (13), 24 (14); NC (4); NC 5AAS (12); NC H_2_O (8). (Ns) for FABP‐2 were the same with the exception of EHS 5AAS 30 (13), 3 (16); EHS H_2_O 24 (14); EXC 5AAS 30 (15), 3 (11); EXC H_2_O 3 (14); NC H_2_O (11).

The kidney, muscle, and liver displayed a distinct response to EHS irrespective of treatment. Plasma levels of BUN were transiently elevated from both EXC and NC groups at 30 min (*p* < 0.0001 for all) (Figure 10B). CK and AST displayed late phase peak responses at 3 h (*p* < 0.05), which returned to baseline at 24 h (Figure 10A,C). No visible differences existed between 5AAS and H_2_O groups in organ associated damage markers.

#### 3.2.7 Gut hormones

Metabolic hormones of the gut including GIP and PYY were not different between treatment groups at any time. Although GIP following EHS was significantly lower in the early phases of recovery (30 min, 3 h) compared to 24 h for both treatment groups (*p* < 0.05). GLP‐1 was below detection for all groups.

#### 3.2.8 Plasma FABP‐2 as an indirect measure of gut permeability

FABP‐2 in EXC mice was virtually identical to the NC groups regardless of the recovery time point or treatment (≈5 ng/mL) (Figure 10D). EHS induced a significant increase in FABP‐2 (≈80 ng/mL) compared to both NC and EXC groups at 30 min; this recovered by 3 h and remained at baseline at 24 h (Figure 10D; *p* < 0.0001 for all groups). No differences in FABP‐2 release were detected between treatment groups at any time point.

## 4 DISCUSSION

The primary finding of this study demonstrates that a 5AAS administered prophylactically has an observable effect following EHS, as demonstrated by abatement of hypothermia depth, length, and the minimal Tc attained (Table 1 & Figure 1). Contrary to our hypotheses, this dose and timing of 5AAS administration did not affect thermoregulation in the heat or the subsequent inflammatory response (Table 1 & Figures 9 and 10). Although it was associated with a reduction in ionic particle permeation (transepithelial conductance) (Figure 2A,B), non‐ionic particle permeation (4 kDa FITC‐dextran) (Figure 2C,D), improved villus length (Figure 3 A–D), increased sodium absorption (Figure 3E) and changes in barrier protein expression pattern (Figures 4, 5, 6, 7). These outcomes suggest improved barrier formation, all occurring at 30 min into the recovery period following EHS. The results of this study suggest that 5AAS mitigates the severity of the gastrointestinal mucosal injury as observed by histomorphometric measurements (Figure 3A–D), increased electrolyte absorption (Figure 3E) and improved barrier function. The results also carry implications for understanding the underlying mechanisms for improved thermoregulatory responses such as hypothermia depth, length, and the minimal Tc. These studies agree with our previous observation where 5AAS was shown to reverse radiation‐induced functional and structural disruption of the intestinal barrier in mice, where 5AAS was administered by oral gavage following radiation exposure (Gupta et al., 2020).

This is the first study to demonstrate that a 5AAS mitigates the extent of hypothermia following EHS in mice. Surprisingly, this effect was not related to major differences in circulating cytokine response, or differences in circulating markers of organ damage. Previous studies have implied cytokines of the innate immune response as potential mediators of regulated hypothermia in mice (Leon, 2004). However, we present a significantly altered hypothermic response in 5AAS without differences in cytokine or chemokine responses. Although it is possible that the influence of mediators that affect body temperature, particularly those that mediate hypothermia, for example leukotrienes, are outside of detection range of the inflammatory cytokines measured here (Carlin et al., 2017; Nunes et al., 1991).

This contradicts our previous observations where the select amino acids attenuated the increased conductance, plasma endotoxin and proinflammatory cytokines following radiation exposure (Yin et al., 2014). In radiation‐induced GI‐toxicity studies the select amino acid formulation was given following radiation exposure as daily gavage for a period of 7 days. In the current study, the formulation was given as a single 150 μL oral gavage ~12 h prior to the study. This may explain why 5AAS failed to attenuate the early cytokine response and Tc,max (Figures 9 and 10). Further dosing and scheduling studies may help improve 5AAS response to EHS‐induced alterations in systemic cytokine or chemokine responses.

The extent of hypothermia during recovery has been associated with the magnitude and duration of heat stress exposure as well as the severity of injury. Data demonstrate that while hypothermia may be a protective mechanism, mice that reach lower Tcs have worse outcomes, suggesting some degree of hypothermia mitigation is beneficial (Leon et al., 2005; Leon et al., 2006; van der Linde et al., 1992; Wilkinson et al., 1988). Our current study demonstrates that exercise performance in the heat (distance run, max speed attained) and measures of thermal stress (time to Tc,max, Tc,max, ascending thermal area (TA), descending TA, total TA) were similar between groups, yet the 5AAS solution significantly attenuated the extent of hypothermia during recovery. This suggests that the time spent in hypothermia may be indicative of another stressor other than the extent of heat stress (Carlin et al., 2017), for example substrate availability. Indeed, there are amino acid sensors in the gut that relay the nutrient composition to the brain (Liu et al., 2016), which may play a role in detecting substrate availability and the emergence from hypothermia.

Our secondary finding demonstrated small but important changes in the GI barrier. Tight junctions (TJ) and adherent junction (AJs) provide important adhesive contacts between neighboring cells and regulate permeability of the intestinal barrier to ionic molecules, nonionic small organic solutes, and water through paracellular spaces (Murphy et al., 2013). Decreased villus height (and subsequently the total surface area of absorption) and increased mucosal permeability were observed at 30 min into recovery, suggesting pathogenesis of mucosal injury. Increased transepithelial electrical conductance can enhance translocation of FITC‐dextran following EHS, suggesting enhanced translocation of antigenic substances from the gut lumen into the systemic circulation.

However, instead of supporting the hypothesis that the innate immune cytokine responses are derived from increased gut permeability and subsequent endotoxin release, these data support an alternative hypothesis that the heat stress induced cytokine responses to endotoxin are negligible or absent (Welc et al., 1985). If the cytokine response was only related to leaky gut, the 5AAS given prophylactically may not have been sufficient to overcome the initial cytokine surge, but the specific amino acids could accelerate the speed of recovery from mucosal injury. This notion is supported by the observation that 5AAS treated mice group showed decreased conductance, non‐ionic particle permeation at 30 min, increased sodium absorption at 30 min and 3 h, and increased villus height at 30 min with no significant differences in any of these measurements after 24 h into recovery. We found that EHS expression decreased occludin, claudin‐1 and claudin‐5 (Figures 4, 5 and 7) while increasing the claudin 2 (Figure 6) expression. 5AAS pretreatment increased occludin, claudin‐1, claudin‐5 and decreased claudin‐2 expression (Prasad et al., 2005; Zeissig et al., 2007). Upregulation of barrier‐forming claudins increases transepithelial resistance, whereas upregulation of pore‐forming claudins reduces resistance. Therefore, the reduction in the conductance and non‐ionic particle permeation with 5AAS may account for the changes observed with expression patterns of TJ proteins. Based on representative images, these changes occurred without a change in the expression levels of e‐cadherin in EHS and following treatment, suggesting that e‐cadherin did not play a major role in EHS mediated mucosal injury. Since e‐cadherin is not required for the assembly of TJs and the formation of AJs is required for organization of TJs, the present observations on TJ proteins and e‐cadherin suggest that EHS induced changes mostly occur at the level of TJs, although this should be investigated in the future through more quantitative measures.

Transepithelial conductance and non‐ionic particle permeation are both measures of intestinal paracellular permeability. Transepithelial conductance is the sum of transcellular and paracellular permeability. Paracellular pathway accounts for >90% of transepithelial conductance in a leaky epithelium such as mouse small intestine and is the major contributor to barrier dysfunction (Vidyasagar & MacGregor, 2016). In this study, the increased conductance and FITC‐dextran permeation were observed mostly in the jejunum and not in the ileum, suggesting that EHS‐induced mucosal injury being confined mostly to the leaky regions of the small intestine such as the jejunum. A large body of evidence implies that circulating markers and indicators of the innate immune response (i.e., cytokines and chemokines) are directly related to increases in gut permeability and subsequently the severity of injury in critical illness (Casey et al., 1993; Clark & Coopersmith, 2007; Yin et al., 2016). Here, we show differences in gut permeability as measured by changes in conductance (Figure 2A,B) and FITC‐dextran permeation (Figure 2C,D) between treatment groups at 30 min recovery without changes in the innate immune response or markers of organ damage, suggesting that widespread systemic inflammation is not specifically derived from the gut following EHS. In fact, we were unable to detect bacterial 16S rRNA in the plasma indicating that endotoxin leakage was not responsible for the initiation of the systemic inflammatory response or multi‐organ dysfunction. Although we only examined select time points, it is likely they represent the overall pattern and time course of the innate immune response and organ injury. This suggests that other mechanisms may be responsible for the systemic inflammatory response (Welc et al., 1985).

The direct effect of thermal injury on organ tissues is one potential mechanism that contributes substantially to systemic inflammation (Bischof et al., 1995; Bynum et al., 1978; Kiyatkin & Sharma, 2009; Lim & Mackinnon, 2006). Indeed, the 5AAS and H_2_O groups experienced nearly identical thermal areas and maximal Tcs, suggesting inflammatory responses and organ damage should be similar between groups. Previous investigations suggest that the splanchnic nerves play a strong role in the suppression of the innate immune response; therefore, it is possible that the “inflammatory reflex” suppressed inflammation in both the 5AAS and H_2_O groups to similar levels (Huston, 2012; Martelli et al., 2019). Lastly, skeletal muscle may have a large contribution to the circulating inflammatory response (Pedersen et al., 2001a; Welc et al., 1985). Exercise performance (max speed and distance) and muscle damage were similar between treatment groups supporting the role of the skeletal muscle in the initiation of the systemic inflammatory response (Pedersen et al., 2001b; Pedersen & Febbraio, 2008; Pillon et al., 2013).

### 4.1 Experimental considerations

Timing of the 5AAS administration solution is an experimental consideration for this study. Because oral gavage is stressful to the mouse it was imperative that we administered the solution the evening before the experimental protocol to prevent handling induced increases in Tc. The effects of the 5AAS on gut integrity coincided with the mitigation of hypothermia, suggesting that perhaps more frequent administration of the solution or administration at a time point closer to heat exposure would have revealed additional and/or different beneficial effects that were not demonstrated in this study. Importantly, the phenomenon of hyperthermia induced hypothermia is unique to small rodents. Although hypothermia is thought to promote survival through a reduction in metabolic demand (Gordon, 1993; Leon et al., 2005) and has been demonstrated following a variety of other stressors including hypoxia, caloric restriction, and chemical toxin exposure, its translation to human populations in unknown.

Future experiments should explore timing of solution administration as well as utilize a variety of control solutions. While water serves as an ecologically relevant comparator, this solution does not allow us to determine the independent effects of the caloric, sodium, or potassium loads on EHS outcomes. As sex differences have been previously demonstrated in EHS (Garcia et al., 2018), female mice should be included in future experiments.

## 5 CONCLUSION

We provide novel evidence that a 5AAS pretreatment enhances EHS recovery through the mitigation of hyperthermia induced hypothermia as demonstrated by attenuation of hypothermia depth, length, and the minimal Tc attained, with improvements in villus length, increased net sodium absorption, decreased conductance, non‐ionic particle permeation and changes in TJ expression pattern suggestive of improved intestinal barrier integrity. The 5AAS formulation was shown to increase barrier forming claudins and decrease the expression pattern of pore‐forming claudin‐2 in epithelial membranes of an EHS mouse model, thereby transforming a “leaky’ upper small intestine into a “tighter” barrier. These data suggest that the innate immune response and markers of organ injury are not related to the extent of small intestine permeability but rather a more systemic heat induced cytokine response. These data call for further studies to better understand the role of gut permeability, the systemic inflammatory response and the dosing and scheduling of 5AAS to better mitigate the heat stroke‐induced intestinal dysfunction and systemic alterations.

## AUTHOR CONTRIBUTIONS

*Conception and design*: Michelle A. King, Thomas L. Clanton, Sadasivan Vidyasagar, Shauna M. Ward, Matthew D. Ward, Astrid Grosche. *Acquisition, analysis or interpretation of data*: Michelle A. King, Thomas L. Clanton, Astrid Grosche, Anusree Sasidharan, XiaoDong Xu, Sadasivan Vidyasagar, Jermaine A. Ward, Shauna M. Ward, Mark L. Plamper, Thomas A. Mayer. *Drafting manuscript or critical revisions*: Michelle A. King, Thomas L. Clanton, Astrid Grosche, Anusree Sasidharan, XiaoDong Xu, Sadasivan Vidyasagar, Jermaine A. Ward, Shauna M. Ward, Mark L. Plamper, Thomas A. Mayer.

## ETHICS STATEMENT

All vertebrate animal experiments were performed in accordance with the Animal Welfare Act and the Office of Laboratory Animal Welfare regulations, NIH, USA. The University of Florida Institutional Animal Care and Use Committee approved all animal protocols.

## FUNDING INFORMATION

This work was funded by the United States Army Research Institute of Environmental Medicine and Entrinsic Bioscience, Inc.

## CONFLICT OF INTEREST STATEMENT

Sadasivan Vidyasagar has shares in Entrinsic Bioscience, Inc. All other authors declare that they have no conflict of interest.

## Supporting information


Data S1:
